# Oncolytic Virotherapy Increases the Detection of Microscopic Metastatic Disease at Time of Staging Laparoscopy for Pancreatic Adenocarcinoma

**DOI:** 10.1016/j.ebiom.2016.05.019

**Published:** 2016-05-19

**Authors:** Christopher J. LaRocca, Julia Davydova

**Affiliations:** a Department of Surgery, University of Minnesota, Minneapolis, MN, United States; b Masonic Cancer Center, University of Minnesota, Minneapolis, MN, United States

The field of oncolytic virotherapy has undergone much growth in recent years as improvements in vector design have resulted in augmented selectivity for specific cancer types as well as more potent anti-cancer effects. There is probably no greater evidence of such progress as the recent Food and Drug Administration (FDA) approval of talimogene laherparepvec (T-VEC) for the treatment of melanoma following the published results of the OPTiM study (Andtbacka et al., 2015). As the first FDA-approved oncolytic virotherapy in the United States, it will hopefully serve as a stepping-stone for the development and approval of additional viral-based therapies.

Another important component to the evolution of oncolytic virotherapy has been its use in non-invasive bioimaging. With increasing numbers of vectors entering into trials, the ability to effectively monitor the pharmacokinetics and pharmacodynamics of infection will be critical. Some groups have employed transgenes such as an enhanced green fluorescent protein (eGFP) to analyze fluorescence from their vectors (Adusumilli et al., 2011). Other avenues of investigation have looked into reporter genes encoding membrane transport proteins such as the sodium iodide symporter (NIS) (Miller and Russell, 2016).

One application of these molecular imaging techniques is to use the information that they can provide to augment diagnostic techniques in difficult clinical situations. For example, pancreatic adenocarcinoma has one of the poorest survivals for all types of malignancies. Even with modern chemotherapeutics and clinical care, the overall five year survival rate is only on the order of 6%. For patients with localized disease, surgical resection remains a cornerstone of therapy.

A critical component to care of these patients is ensuring that they are appropriate candidates for surgery, and that they do not have metastatic disease. Staging laparoscopy has the potential for identifying metastatic liver or peritoneal disease in patients who were otherwise thought to have only localized disease on cross sectional imaging (Pisters et al., 2001). This practice has not been universally adopted and there is no standard criteria for selecting the appropriate patients (De Rosa et al., 2016). However, some authors have found a correlation between elevated serum CA 19-9 levels and findings of metastatic disease at the time of laparoscopy, and thereby suggest that these patients may be better candidates to undergo such a procedure (Karachristos et al., 2005).

Cytological analysis performed following staging laparoscopy for patient with pancreatic adenocarcinoma is highly specific, but lacking in sensitivity. This study by Kelly et al. has sought to improve upon conventional cytology by employing a modified herpes virus expressing the eGFP gene to better identify tumor cells in peritoneal washings (Kelly et al., 2016).

The NV1066 virus used in the study is an attenuated, replication-competent oncolytic herpes simplex virus type 1 (HSV-1). Additionally, the eGFP transgene was inserted into the virus genome and is under the control of the cytomegalovirus promoter. Previously, this vector has been demonstrated to have the ability to detect as few as one cancer cell in a background of one million normal cells (Adusumilli et al., 2011).

Eighty-two patients with proven pancreatic adenocarcinoma were enrolled in the study. Peritoneal washing were obtained during diagnostic laparoscopy and then analyzed by conventional cytology and HSV-mediated fluorescence detection. In the cytology arm, the sensitivities for detecting any metastatic disease and for the detection of peritoneal metastases were 44% and 89% respectively. In contrast, the viral-mediated detection arm had sensitivities of 94% and 100% respectively.

For those patients with negative conventional cytology who went on to curative-intent surgery, there was a statistically significant (p = 0.01) decrease in recurrence free survival for those patients who were eGFP positive (6.5 months) compared to eGFP negative patients (12.2 months).

As the authors have explained, many patients with pancreatic cancer undergo staging laparoscopy and peritoneal washings, only to have a false-negative result upon cytological analysis. In this manuscript, they have clearly demonstrated that their modified herpes virus was effective at infecting and replicating within pancreatic adenocarcinoma cells from many different patients. This application of oncolytic virus-based imaging outperforms conventional cytology approaches as evidence by the markedly increased diagnostic sensitivity. This type of information can and will be helpful in guiding clinical decision making for patients with pancreatic adenocarcinoma.

Given the results of this study, it is clear that this herpes simplex oncolytic virus deserves further testing in larger trials so that it can continue to move towards FDA approval for clinical use. The potential applications of this vector extend beyond pancreatic cancer, and we would also anticipate that the authors will be pursuing additional studies analyzing peritoneal washings for other malignancies, such as gastric cancer.
